# Refusal of Guideline-Recommended Adjuvant Therapy in Oral Squamous Cell Carcinoma Is Associated with Less Favorable Survival Outcomes

**DOI:** 10.3390/cancers18111795

**Published:** 2026-06-01

**Authors:** Lennart Johannes Gruber, Johanna Zirbes-Klingenhäger, Georg Hoene, Frederik Holdorf, Martin Leu, Rainer Laskawi, Henning Schliephake, Susanne Wolfer

**Affiliations:** 1 Department of Oral and Maxillofacial Surgery, University Medical Center Göttingen, Georg-August-University, 37075 Göttingen, Germanyfrederik.holdorf@med.uni-goettingen.de (F.H.); susanne.wolfer@med.uni-goettingen.de (S.W.); 2 Department of Radiotherapy and Radiation Oncology, University Medical Center Göttingen, Georg-August-University, 37075 Göttingen, Germany; 3 Department of Otorhinolaryngology, Head and Neck Surgery, University Medical Center Göttingen, Georg-August-University, 37075 Göttingen, Germany

**Keywords:** oral squamous cell carcinoma, adjuvant therapy, treatment refusal, guideline adherence, survival outcomes, risk factors

## Abstract

Background/Objectives: Guideline-recommended adjuvant therapy, including radiotherapy alone or concurrent chemoradiotherapy, is an established component of treatment for patients with high-risk oral squamous cell carcinoma (OSCC). However, a considerable proportion of patients (30.7%) refuse postoperative treatment despite a clear guideline-based recommendation. Data on determinants of treatment refusal and its association with oncologic outcomes remain limited. Methods: This retrospective single-center cohort study included 88 patients with histologically confirmed OSCC and a clear indication for adjuvant therapy between 2015 and 2021. Overall survival (OS) and disease-free survival (DFS) were estimated using the Kaplan–Meier method. Group comparisons were performed using Breslow tests to account for potential early differences in survival. Exploratory Cox proportional hazards models were calculated for OS to assess the association between completion of adjuvant therapy and survival after limited adjustment for clinically relevant covariates. Results: Refusal of indicated adjuvant therapy occurred in 27 of 88 patients (30.7%) and was significantly associated with older age (*p* = 0.002) and female sex (*p* = 0.002). In unadjusted Kaplan–Meier analyses, patients who refused adjuvant therapy showed shorter median OS compared with those who completed treatment, although the difference did not reach statistical significance (*p* = 0.155). In exploratory Cox regression analyses, completion of adjuvant therapy was associated with a reduced risk of death after adjustment for age and sex (HR 0.22, 95% CI 0.09–0.54; *p* = 0.001). In the overall cohort, no statistically significant difference in DFS was observed according to adjuvant therapy status (*p* = 0.129). However, exploratory subgroup analyses suggested that DFS differences according to adjuvant therapy status were more apparent in patients with advanced primary tumors (pT3/4; *p* = 0.032) and those with more extensive nodal disease (pN2/3; *p* = 0.019). Conclusions: Refusal of guideline-recommended adjuvant therapy is frequent in patients with high-risk OSCC and was associated with less favorable survival patterns in this cohort. Given the retrospective design, limited sample size, and exploratory nature of the analyses, these findings should be interpreted as associative rather than causal. Nevertheless, they highlight the potential clinical relevance of treatment adherence and support the importance of patient-centered counseling to improve uptake of guideline-recommended postoperative therapy.

## Simple Summary

Adjuvant therapy, including radiotherapy alone or concurrent chemoradiotherapy, is commonly recommended after surgery for patients with advanced oral squamous cell carcinoma to reduce the risk of tumor recurrence and disease progression. However, a substantial proportion of patients refuse the recommended treatment. Data on the frequency of treatment refusal and its association with survival outcomes remain limited. In this study, we analyzed how often patients refused guideline-recommended adjuvant therapy, which factors were associated with refusal, and how this was related to survival outcomes. We found that refusal was more frequent in older patients and women and was associated with less favorable survival patterns, particularly in patients with advanced disease. Because this was a retrospective single-center study, the findings should be interpreted cautiously and do not prove causality. Nevertheless, they underline the potential clinical impact of treatment refusal and the importance of strategies to improve adherence to recommended postoperative therapy.

## 1. Introduction

Oral squamous cell carcinoma (OSCC) represents one of the most common malignancies of the head and neck region and continues to be associated with an unfavorable prognosis, particularly in advanced disease stages [1]. Despite improvements in diagnostic techniques and multidisciplinary treatment strategies, overall survival (OS) rates for OSCC have shown only modest improvement over recent decades [2,3]. Most patients are diagnosed with locally advanced disease, emphasizing the clinical relevance of optimized postoperative management [4]. Surgical resection remains the cornerstone of curative treatment for OSCC. In patients with advanced primary tumor stage, lymph node metastases, extracapsular extension, perineural invasion, lymphovascular invasion, or positive or close resection margins, postoperative radiotherapy alone or concurrent chemoradiotherapy is recommended as a guideline-based standard of care [5,6,7,8]. In the present study, a clear guideline-based indication was operationally defined as a definitive interdisciplinary tumor board recommendation for postoperative adjuvant therapy based on adverse pathological criteria, including advanced primary tumor stage (pT3/pT4), advanced nodal disease (pN2/pN3), extracapsular extension, positive or close resection margins, perineural invasion, lymphovascular invasion, or other high-risk constellations considered clinically relevant by the tumor board.

Multiple studies and clinical guidelines have demonstrated that adjuvant therapy improves locoregional control and survival outcomes in these high-risk patients [5,9].

However, adherence to guideline-recommended adjuvant treatment in routine clinical practice is not universal. A substantial proportion of patients refuse recommended adjuvant therapy despite a clear oncologic indication [10]. This poses a substantial challenge, as refusal of adjuvant therapy may compromise oncologic outcomes and undermine the benefits of an otherwise curative surgical approach [10,11]. While patient-related factors such as age, comorbidities, and treatment burden are often presumed to influence this decision, recent analyses of OSCC cohorts have demonstrated that refusal of recommended adjuvant therapy is primarily associated with patient-related rather than tumor-related characteristics [12]. Data specifically addressing predictors and oncologic consequences of adjuvant therapy refusal in patients with OSCC remain scarce and are largely derived from small or heterogeneous cohorts [10,12].

Previous studies investigating outcomes of OSCC have largely focused on treatment efficacy in patients who completed multimodal therapy, whereas patients who refused recommended adjuvant treatment have frequently been underrepresented or excluded from analysis [13,14]. As a result, the prognostic impact of adjuvant therapy refusal remains insufficiently characterized, particularly in homogeneous cohorts with a uniform indication for postoperative treatment. Moreover, data on disease-free survival (DFS) in this context are scarce [10,11]. The present study aims to address this gap by analyzing a retrospective cohort of patients with OSCC who had a guideline-based indication for adjuvant therapy.

The primary objective was to examine the association between refusal of indicated adjuvant therapy and oncologic outcomes, including OS and DFS. Secondary objectives included the identification of patient- and tumor-related factors associated with refusal of adjuvant therapy. By focusing on a well-defined high-risk population, this study seeks to provide clinically relevant insight into treatment adherence and its potential association with oncologic outcomes in OSCC.

We hypothesized that refusal of guideline-recommended adjuvant therapy would be associated with less favorable OS and DFS and that refusal would be more strongly related to patient-related than tumor-related factors.

## 2. Materials and Methods

### 2.1. Study Design and Patient Selection

This retrospective single-center study was conducted at the Department of Oral and Maxillofacial Surgery, University Medical Center Göttingen, and covered the period from 1 January 2015 to 30 August 2021 (cut-off date). The study was conducted in accordance with the Declaration of Helsinki and approved by the Ethics Committee of the University Medical Center Göttingen (protocol code 8/2/22, approval date: 8 February 2022).

Potential cases were identified through the institutional medical controlling database using ICD-10 codes for malignant neoplasms of the oral cavity (C02–C06), including of the tongue, floor of the mouth, buccal mucosa, palate, and gingiva of the alveolar process. This search yielded an initial cohort of 316 patients. All cases subsequently underwent detailed chart review to verify diagnosis, histopathology, and treatment characteristics. Patients without histologically confirmed OSCC, with miscoded diagnoses, or with tumors originating from the lip, oropharynx, nasopharynx, or hypopharynx were excluded. Predefined clinical and pathological inclusion criteria were then applied to identify patients with a guideline-based indication for postoperative adjuvant therapy. In this study, adjuvant therapy refers to postoperative radiotherapy alone or concurrent chemoradiotherapy, depending on the individual risk profile and interdisciplinary tumor board recommendation. Guideline-based indications for postoperative adjuvant therapy were defined according to contemporary national and international guideline recommendations (including NCCN and German S3 guideline recommendations) applicable during the study period and were confirmed by the institutional interdisciplinary head and neck tumor board. Indications included adverse pathological features such as advanced primary tumor stage, nodal involvement, extracapsular extension, positive or close surgical margins, perineural invasion, lymphovascular invasion, and other high-risk constellations considered relevant by the tumor board. Patients were classified as having a definitive indication when adjuvant treatment was clearly recommended by the tumor board on the basis of these criteria. Patients with optional or facultative recommendations were not included in the survival analyses.

**Figure 1:**
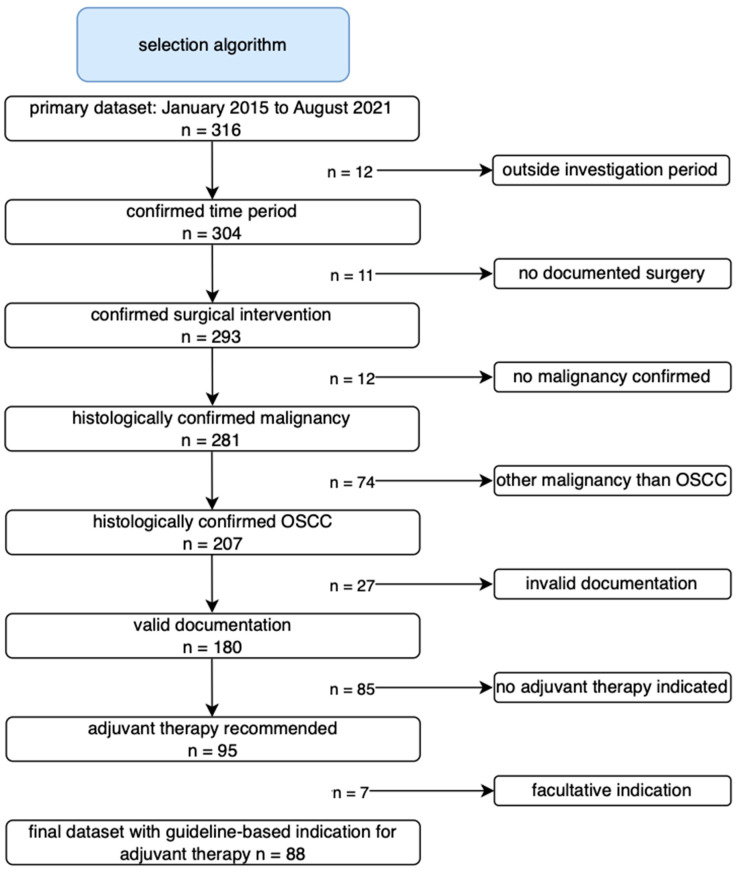
Flowchart of patient selection. From 316 cases retrieved using ICD-10 codes (C02–C06) for oral cavity malignancies, patients with histologically confirmed oral squamous cell carcinoma and a guideline-based indication for adjuvant therapy formed the final study cohort (n = 88).

After application of all eligibility criteria, 95 patients constituted the final study cohort. Of these, 88 patients had a definitive indication for guideline-recommended adjuvant therapy, while seven patients received a recommendation for optional adjuvant radiotherapy. Survival analyses were restricted to patients with a definitive guideline-based indication for adjuvant therapy (n = 88). Among patients with a definitive indication, 61 received the recommended therapy and 27 refused treatment. The stepwise patient selection process is illustrated in Figure 1.

### 2.2. Data Collection and Clinical Variables

Clinical and pathological data were extracted from patient records, including interdisciplinary tumor board reports, pathology reports, operative reports, discharge letters, and radiotherapy documentation. Recorded variables included age at diagnosis, sex, tumor localization, ICD-10 classification, date of surgery, and follow-up information. Sex was recorded as documented in the clinical records; no separate gender-related variables were available for analysis. The tumor site categories shown in Table 1 were derived from ICD-10 codes. In cases with involvement of multiple subsites, tumors were assigned to a single category based on the documented ICD-10 classification. Pathological parameters comprised TNM classification, depth of invasion, tumor grading, resection margin status, perineural invasion, lymphovascular invasion, and extracapsular extension of lymph node metastases. Information regarding neck dissection and extent of lymph node surgery was obtained from surgical reports. Tumor board documentation provided information on the indication for postoperative radiotherapy alone, concurrent chemoradiotherapy, or optional adjuvant therapy. For patients undergoing adjuvant treatment, treatment modality and treatment dates were recorded. Adjuvant treatment was delivered according to institutional standards and contemporary guideline recommendations during the study period. Detailed analyses of radiotherapy dose, fractionation schedules, or comparative evaluation of radiotherapy versus chemoradiotherapy were not performed because the objective of the present study was to analyze completion versus refusal of the recommended adjuvant treatment strategy rather than treatment-specific effectiveness. Reasons for refusal and patient-level factors potentially influencing treatment decisions, such as comorbidities, frailty, performance status, socioeconomic factors, or psychosocial factors, were not available as standardized structured variables and therefore could not be analyzed systematically.

### 2.3. Outcome Definitions

OS was defined as the time from initial diagnosis to death from any cause or last documented patient contact. Recurrence events included both locoregional recurrence and distant metastatic disease where documented. DFS was defined as the time from diagnosis to first documented recurrence or disease-related death. Patients without an event were censored at the date of last documented patient contact.

### 2.4. Statistical Analysis

Statistical analyses were performed using R software (Version 4.5.2; R Foundation for Statistical Computing, Vienna, Austria). Descriptive statistics and Kaplan–Meier survival analyses were conducted. OS and DFS were estimated using the Kaplan–Meier method, and median survival times as well as 2-year and 5-year survival rates were calculated.

Differences between survival curves were assessed using Breslow (generalized Wilcoxon) tests, which place greater emphasis on early survival differences and were considered appropriate because early recurrence and mortality events were regarded as clinically relevant in this high-risk cohort. Associations between categorical variables, including determinants of refusal of adjuvant therapy, were analyzed using Fisher’s exact test. Statistical significance was defined as a two-sided *p*-value ≤ 0.05. No correction for multiple testing was applied because the analyses were considered exploratory and hypothesis-generating rather than confirmatory.

Additional analyses of OS were performed using Cox proportional hazards regression models. Univariate and multivariable models were calculated to assess the association between adjuvant therapy and OS. Covariates were selected a priori based on clinical relevance and the number of observed events to reduce the risk of overfitting. Age (per 10-year increase) and sex were included as baseline variables, and nodal status (pN0/1 vs. pN2/3) was evaluated in an additional model. Hazard ratios (HRs) with 95% confidence intervals (CIs) were reported. The proportional hazards assumption was assessed using Schoenfeld residuals. Multivariable Cox regression analyses were restricted to patients with complete data for all included covariates (complete-case analysis). Missingness was low for most routinely documented pathological variables; however, selected variables, particularly depth of invasion, were incompletely available. Broader multivariable adjustment was not pursued because of the limited number of observed events and the resulting risk of model overfitting. In addition, clinically relevant patient-level variables, including comorbidity burden, frailty, ECOG performance status, psychosocial factors, socioeconomic factors, social support, treatment preferences, and reasons for treatment refusal, were not available as standardized structured variables. Given the limited number of events, these analyses were considered exploratory. Potentially relevant confounders such as comorbidities, frailty, performance status, psychosocial factors, and socioeconomic variables could not be included because these data were not documented consistently enough for reliable analysis in this retrospective cohort. Because adjuvant therapy was initiated postoperatively and required survival until treatment initiation and completion, immortal time bias cannot be excluded completely. Given the retrospective design and limited sample size, no formal landmark or time-dependent sensitivity analyses were performed. Accordingly, all survival analyses should be interpreted as exploratory and associative rather than causal.

Cox regression modeling was not performed for DFS because the proportional hazards assumption was violated, indicating time-dependent effects that could not be adequately captured by the model. In addition, the number of events relative to the available covariates was limited. Alternative time-dependent Cox models were considered; however, these approaches were not pursued because the limited subgroup sizes and event numbers were considered insufficient for robust and reliable modeling. Therefore, DFS analyses were restricted to Kaplan–Meier estimates and subgroup comparisons and should be interpreted as exploratory and descriptive rather than as providing independent estimates of effect.

## 3. Results

### 3.1. Patient Characteristics

A total of 95 patients with OSCC met the inclusion criteria. Among these, 88 patients had a clear guideline-based indication for adjuvant therapy and were included in the survival analyses (Figure 1). Adjuvant therapy comprised postoperative radiotherapy alone or concurrent chemoradiotherapy according to the individual risk profile and tumor board recommendation. Among the 61 patients who received adjuvant therapy, 28 received postoperative radiotherapy alone and 33 received concurrent chemoradiotherapy. Concurrent systemic treatment consisted predominantly of cisplatin-based regimens. In this cohort, the majority of patients were male (n = 56, 63.6%) and the mean age was 66 ± 11 years at the time of diagnosis. The age distribution peaked in the sixth and seventh decades of life. Tumors were most frequently located in the floor of the mouth and mandibular gingiva, followed by the tongue and buccal mucosa (Table 1). Advanced disease was common, with UICC stage IV diagnosed in 60 patients (68.2%). Lymph node metastases were present in 66 patients (75.0%), and extracapsular extension occurred in 30 patients (34.1%). Microscopically positive resection margins (R1) were identified in 10 patients (11.4%). A total of 51 DFS events were recorded, including 38 recurrences (43.2%) and 13 deaths (14.8%).

### 3.2. Determinants of Adjuvant Radiotherapy Refusal

Among patients with a clear indication for adjuvant therapy (n = 88), 27 patients (30.7%) refused the recommended treatment. Refusal of indicated adjuvant therapy was significantly associated with patient age and sex (Table 2 and Table 3). Refusal rates increased with age (*p* = 0.002), with the highest proportion observed in patients aged 80–89 years (11 of 15 patients, 73.3%; Table 2). Female patients refused adjuvant therapy more frequently than male patients (16 of 32 (50.0%) vs. 11 of 56 (19.6%), *p* = 0.002; Table 3). Tumor-related variables, including pT classification and pN classification, were not significantly associated with refusal of adjuvant therapy among patients with available staging data (Table 4). Because reasons for refusal and comorbidity- or frailty-related variables were not consistently documented, these associations should be interpreted descriptively and do not allow conclusions regarding the underlying individual decision-making process.

### 3.3. Overall Survival

**Figure 2:**
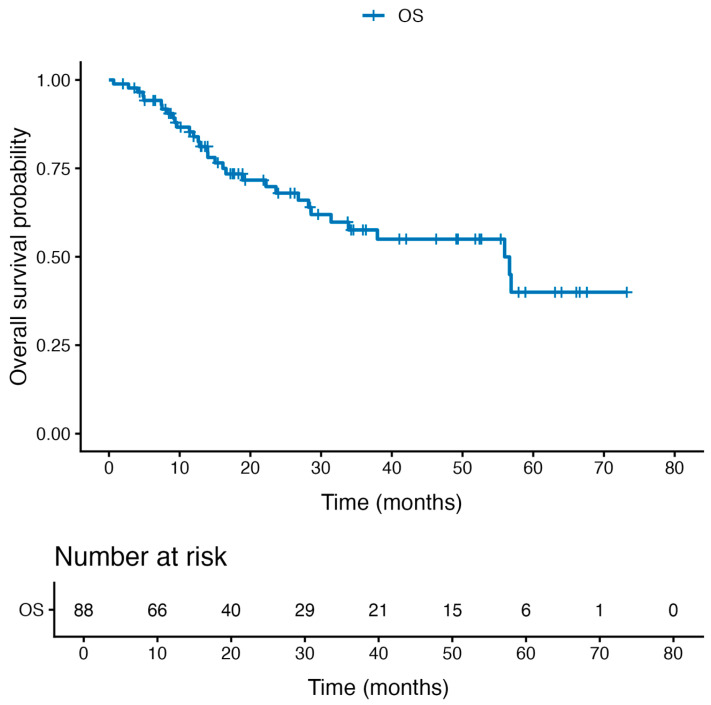
Overall survival of patients with a guideline-based indication for adjuvant therapy (n = 88). Kaplan–Meier estimates of overall survival for the entire study cohort. Tick marks indicate censored observations. Median overall survival was 33 months, and the 5-year overall survival rate was 33%.

**Figure 3:**
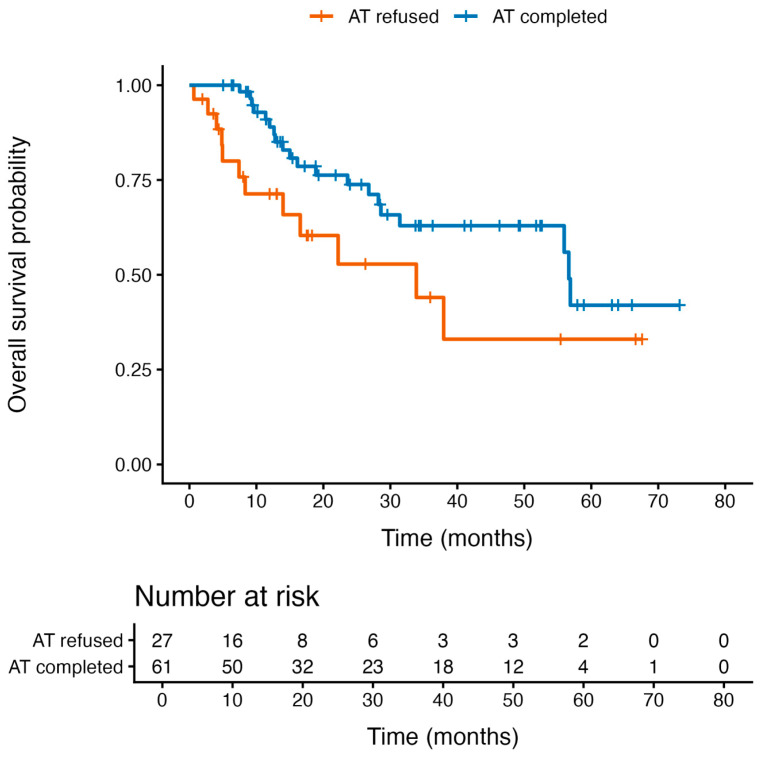
Overall survival according to completion or refusal of adjuvant therapy (n = 88). Kaplan–Meier estimates of overall survival stratified by completion or refusal of guideline-recommended adjuvant therapy (AT). Tick marks indicate censored observations. The difference between groups did not reach statistical significance (Breslow test, *p* = 0.155).

**Figure 4:**
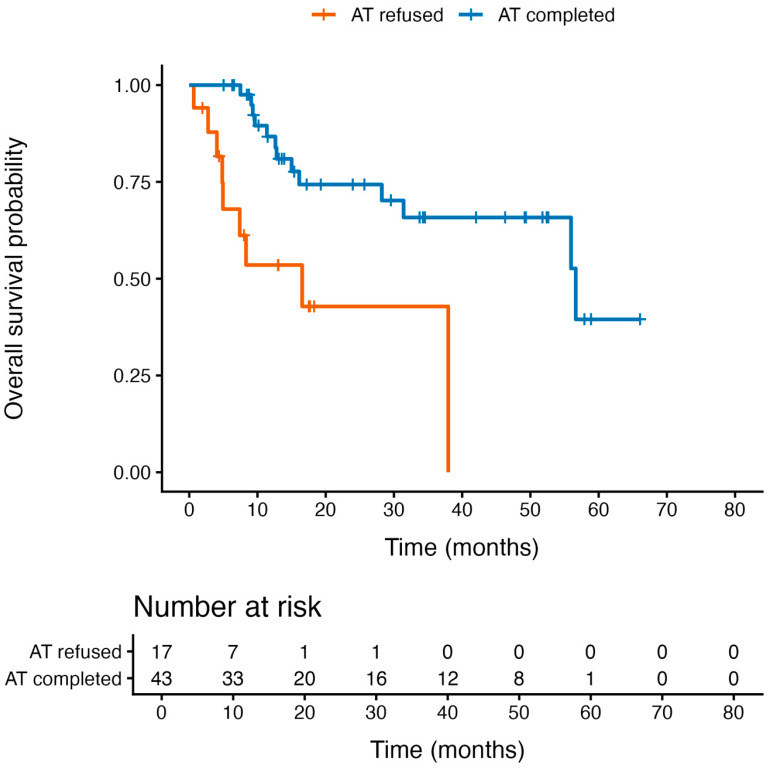
Overall survival in patients with advanced primary tumor stage (pT3/pT4) according to adjuvant therapy status (n = 60). Kaplan–Meier estimates of overall survival in patients with pT3 or pT4 tumors stratified by completion or refusal of adjuvant therapy (AT). Tick marks indicate censored observations. The difference between groups did not reach statistical significance (Breslow test, *p* = 0.074).

**Figure 5:**
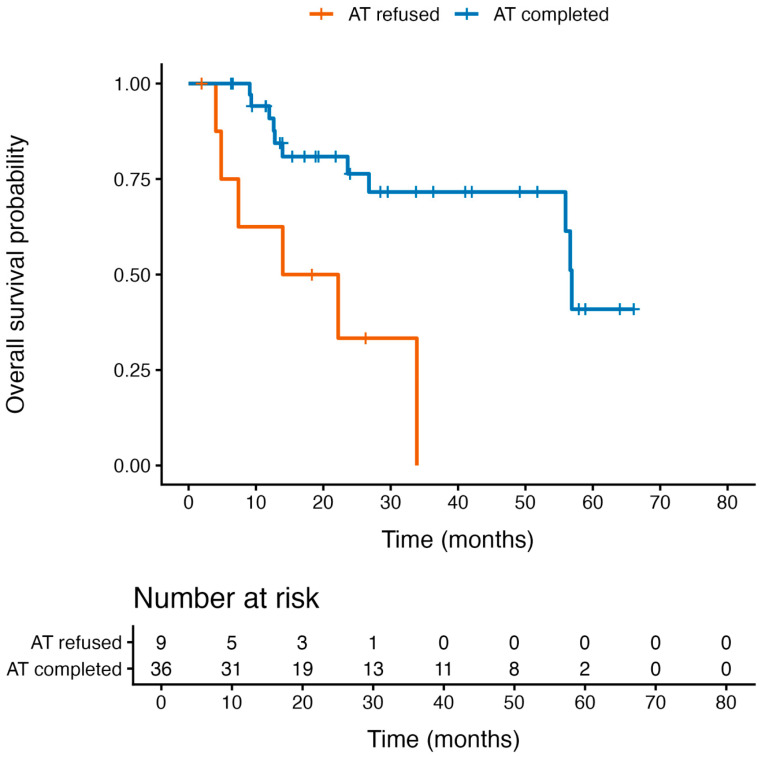
Overall survival in patients with advanced nodal disease (pN2/pN3) according to adjuvant therapy status (n = 45). Kaplan–Meier estimates of overall survival in patients with pN2 or pN3 nodal involvement stratified by completion or refusal of adjuvant therapy (AT). Tick marks indicate censored observations. The difference between groups did not reach statistical significance (Breslow test, *p* = 0.074).

OS was analyzed in patients with a clear indication for adjuvant therapy (n = 88). During follow-up, 32 patients (36.4%) died. The estimated 5-year OS rate was 33%, with a median OS of 33 months (Figure 2). Patients who completed adjuvant therapy demonstrated a longer median OS compared with those who refused the therapy (56.6 vs. 33.8 months; Table 5). However, this difference did not reach statistical significance in unadjusted Kaplan–Meier analysis (Breslow test, *p* = 0.155; Figure 3). Subgroup analyses showed directionally consistent but exploratory trends favoring completion of adjuvant therapy in patients with advanced disease. Among patients with pT3/4 tumors, completion of adjuvant therapy was associated with longer median OS compared with refusal of adjuvant therapy (56.6 vs. 16.5 months), although the difference did not reach statistical significance (Breslow test, *p* = 0.074; Figure 4). Similarly, in patients with pN2/3 nodal disease, completion of adjuvant therapy was associated with longer median OS (56.8 vs. 18.1 months; Breslow test, *p* = 0.074; Figure 5). These subgroup findings were considered exploratory and were not interpreted as confirmatory evidence.

To further evaluate the association between adjuvant therapy and OS, Cox proportional hazards regression analyses were performed (Table 6). In univariate analysis including all patients (n = 88), completion of adjuvant therapy was associated with a reduced risk of death that did not reach statistical significance (HR 0.50, 95% CI 0.24–1.02; *p* = 0.055). In multivariable Cox regression analysis adjusted for age and sex (complete cases, n = 66, events = 32), completion of adjuvant therapy was associated with a significantly reduced risk of death (HR 0.22, 95% CI 0.09–0.54; *p* = 0.001). This association remained consistent after additional adjustment for nodal status (HR 0.23, 95% CI 0.09–0.57; *p* = 0.002). The proportional hazards assumption was met for all models. Given the retrospective design, complete-case restriction, limited number of events, and lack of adjustment for comorbidity, frailty, performance status, and psychosocial variables, these Cox models should be interpreted as exploratory analyses of association rather than evidence of a causal treatment effect.

### 3.4. Prognostic Factors for Overall Survival

In univariate analyses, positive resection margins (R1) were associated with inferior OS (*p* = 0.025). Patients with R1 status demonstrated shorter survival compared with those who achieved R0 resection. Tumor invasion depth was also associated with OS. Patients with an invasion depth greater than 10 mm had worse survival estimates than those with an invasion depth of 10 mm or less (*p* = 0.032). At 24 months, the estimated OS was 49.5% in patients with invasion depth >10 mm compared with 80.1% in patients with less invasion depth. Because depth of invasion was not available for all patients, these variables were not included in multivariable models and should be interpreted with caution. These analyses were exploratory and were used to contextualize established prognostic factors within the present cohort rather than to provide independent prognostic estimates.

### 3.5. Disease-Free Survival

**Figure 6:**
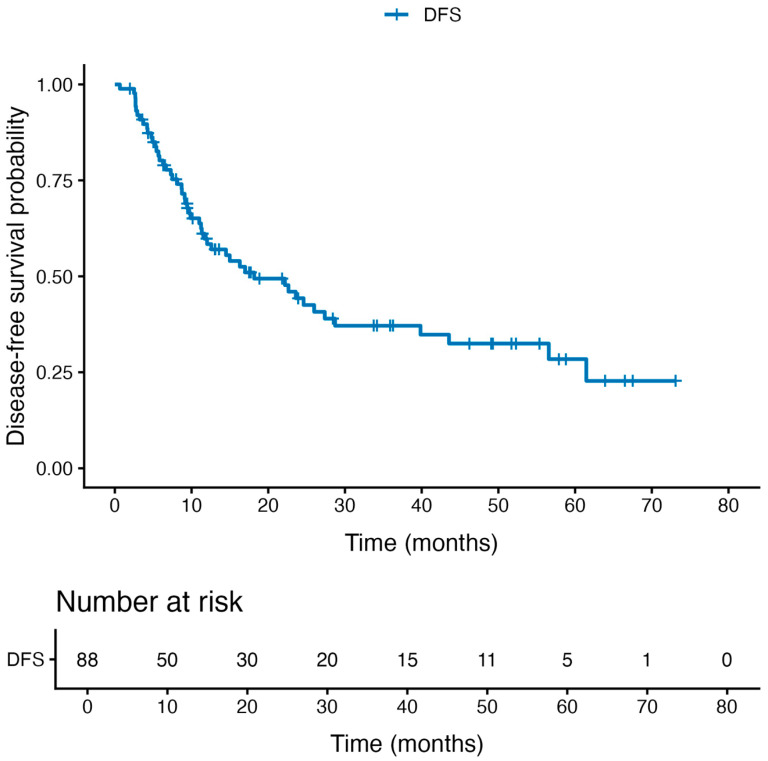
Disease-free survival of patients with a guideline-based indication for adjuvant therapy (n = 88). Kaplan–Meier estimates of disease-free survival for the entire study cohort. Tick marks indicate censored observations.

**Figure 7:**
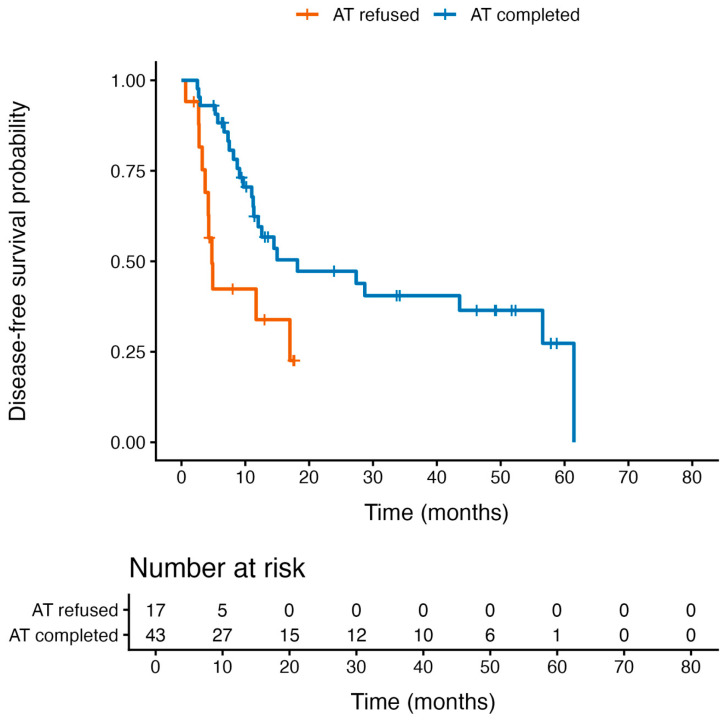
Disease-free survival in patients with advanced primary tumor stage (pT3/pT4) according to adjuvant therapy status (n = 60). Kaplan–Meier estimates of disease-free survival in patients with pT3 or pT4 tumors stratified by completion or refusal of adjuvant therapy (AT). Tick marks indicate censored observations. The difference between groups reached nominal statistical significance in this exploratory subgroup analysis (Breslow test, *p* = 0.032).

DFS was analyzed in patients with a clear indication for adjuvant therapy (n = 88). The estimated 5-year DFS rate was 36.5%, with a median DFS of 33 months (Figure 6). In unadjusted Kaplan–Meier analysis, no statistically significant difference in DFS was observed between patients who completed adjuvant therapy and those who refused treatment (*p* = 0.129). Exploratory subgroup analyses showed more pronounced DFS differences in patients with advanced disease. Patients with advanced primary tumors (pT3/4) showed longer DFS estimates when adjuvant therapy was completed compared with those who refused the treatment in descriptive exploratory subgroup analysis (*p* = 0.032; Figure 7). The median DFS was 55 months in the adjuvant therapy group, compared with 11 months in the group of patients who refused adjuvant therapy.

**Figure 8:**
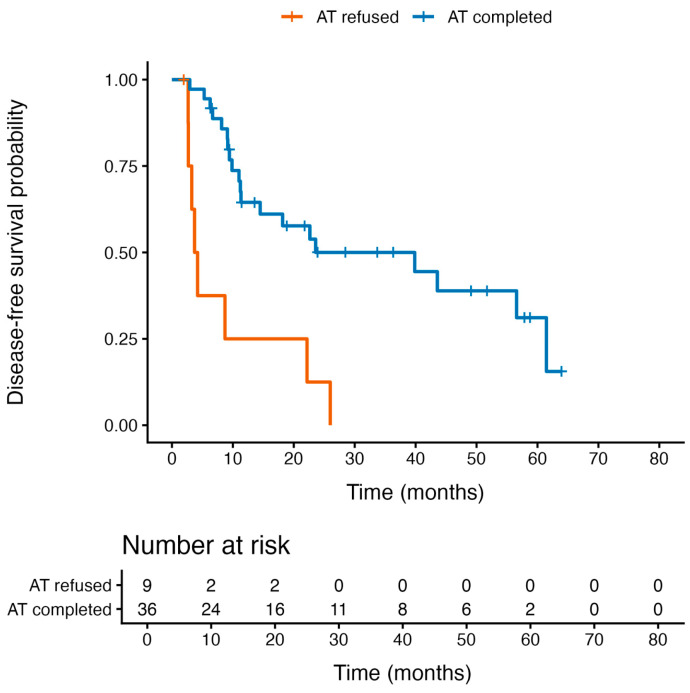
Disease-free survival in patients with advanced nodal disease (pN2/pN3) according to adjuvant therapy status (n = 45). Kaplan–Meier estimates of disease-free survival in patients with pN2 or pN3 nodal involvement stratified by completion or refusal of adjuvant therapy (AT). Tick marks indicate censored observations. The difference between groups reached nominal statistical significance in exploratory subgroup analysis (Breslow test, *p* = 0.019).

Similarly, in patients with advanced nodal disease (pN2/3), completion of adjuvant therapy was associated with longer DFS estimates in descriptive exploratory subgroup analysis (*p* = 0.019; Figure 8). Patients who completed adjuvant therapy showed an estimated 2-year DFS rate of 56.7% and a median DFS of 55 months, whereas patients who refused adjuvant therapy had an estimated 2-year DFS rate of 29.6% and a median DFS of 8 months. Given the limited sample size, the number of events, and the exploratory nature of these subgroup analyses, no adjustment for multiple testing was applied, as these analyses were not designed to provide confirmatory evidence and correction methods may substantially reduce statistical power in small cohorts. Accordingly, these findings should be interpreted as exploratory and descriptive rather than confirmatory. No independent DFS effect estimates were calculated because proportional hazards assumptions were not met and subgroup sizes were insufficient for robust time-dependent modeling. Therefore, *p*-values from DFS subgroup analyses should be interpreted descriptively and not as confirmatory evidence of an independent treatment effect.

In contrast, no statistically significant differences in DFS were observed among patients with lower-risk disease (pT1/2 or pN0/1).

## 4. Discussion

This retrospective study evaluated the clinical relevance of refusal of guideline-recommended adjuvant therapy in patients with OSCC. In this study, adjuvant therapy comprised postoperative radiotherapy alone or concurrent chemoradiotherapy according to the individual pathological risk profile and tumor board recommendation. The principal finding is that refusal of adjuvant therapy was common in this high-risk cohort and was consistently associated with less favorable survival patterns, reflected by shorter median OS and DFS, although not all comparisons reached statistical significance. Given the retrospective single-center design, the limited sample size, and the exploratory nature of the analyses, these findings should be interpreted as hypothesis-generating and associative rather than causal.

In the present cohort, 27 of 88 patients (30.7%) refused guideline-recommended adjuvant therapy. This proportion exceeds refusal rates reported in other oncologic studies, where postoperative radiotherapy refusal was described as uncommon [15], but is in line with more recent investigations in OSCC oncology [10]. Population-based analyses have consistently shown that refusal of indicated cancer treatment is uncommon but associated with inferior survival outcomes [16]. Similar findings have been reported in head and neck cancer cohorts, where refusal of postoperative radiotherapy was associated with increased mortality [11]. More recent analyses focusing specifically on oral cavity cancer further support the association between refusal of indicated adjuvant therapy and worse oncologic outcomes [10,12,17]. The comparatively high refusal rate observed in the present cohort may partly reflect referral patterns, institutional case mix, and the selected high-risk population with a definitive tumor board recommendation for adjuvant treatment. Therefore, direct comparison with registry-based cohorts should be made with caution.

Building on these findings, the present analysis specifically examines determinants of refusal within a well-defined cohort of patients with definitive tumor board-based indications for postoperative adjuvant therapy. This design allowed a focused analysis of treatment refusal among patients for whom postoperative adjuvant treatment was clearly recommended. However, it does not eliminate confounding by indication, particularly because treatment modality and treatment completion are closely related to baseline pathological risk and patient-level factors.

Although the difference in OS between patients with completion of adjuvant therapy and those who refused treatment did not reach statistical significance in unadjusted Kaplan–Meier analysis (*p* = 0.155), the observed difference in median OS (56.6 vs. 33.8 months) suggests a clinically relevant effect size. Exploratory Cox regression analyses demonstrated a consistent association between completion of adjuvant therapy and reduced mortality risk, which remained statistically significant after adjustment for age and sex. However, these findings should be interpreted with caution because residual confounding and limited event numbers cannot be excluded. The discrepancy between unadjusted Kaplan–Meier analyses and adjusted Cox regression results may reflect baseline differences between groups, particularly age and sex, which were associated with both treatment allocation and survival. In addition, unmeasured patient-level factors and limited multivariable adjustment may have contributed to model instability. Accordingly, the Cox regression analyses should be interpreted as exploratory analyses supporting the overall direction of the observed survival pattern rather than as evidence of an independent treatment effect.

A similar pattern was observed for DFS, with shorter recurrence-free intervals among patients who refused adjuvant therapy. However, no statistically significant difference in DFS was observed in the overall cohort. In descriptive exploratory subgroup analyses, DFS differences appeared more pronounced among patients with advanced tumor stage (pT3/4, n = 60) and advanced nodal disease (pN2/3, n = 45) who completed adjuvant therapy. These subgroup findings should be interpreted with caution because subgroup sizes and event numbers were limited, no correction for multiple testing was applied, and no independent DFS effect estimates could be calculated because the proportional hazards assumption was not met. Accordingly, these analyses should be regarded as descriptive and hypothesis-generating rather than confirmatory.

Nevertheless, the observed pattern is biologically and clinically plausible, as the role of postoperative radiotherapy-based treatment is well established in patients with adverse pathological features, including advanced tumor stage, nodal involvement, extracapsular extension, and positive or close margins [5,6,8]. These findings are consistent with current treatment guidelines, which emphasize the role of adjuvant treatment, particularly in patients with advanced tumor stage and high-risk features, while the magnitude of benefit in lower-risk constellations such as pN1 disease without additional risk factors remains less clearly defined [18,19].

A further important consideration is that patients in the present study received either postoperative radiotherapy alone or concurrent chemoradiotherapy according to their individual pathological risk profile. Concurrent chemoradiotherapy was more frequently used in patients with high-risk pathological features. Separate survival analyses comparing radiotherapy alone with chemoradiotherapy were therefore not performed, because subgroup sizes were limited and treatment modality was strongly linked to baseline risk characteristics. Such analyses would have been highly susceptible to confounding by indication and could have produced unstable or potentially misleading estimates. Instead, treatment was analyzed according to completion versus refusal of the guideline-recommended adjuvant treatment strategy.

Refusal of adjuvant therapy was significantly associated with patient-related factors, specifically age and sex. Higher refusal rates were observed in elderly patients, particularly those aged 80 years and older. Age-dependent underutilization of adjuvant oncologic treatment has been reported across multiple cancer entities and is frequently attributed to comorbidities, functional decline, concerns about treatment-related toxicity, and limited perceived benefit [20]. However, the present study did not include systematic assessment of comorbidity burden, frailty, ECOG performance status, social support, socioeconomic status, or treatment-related preferences. As reasons for refusal were not systematically documented, these interpretations remain speculative but are consistent with findings from studies on treatment refusal in oncology [16,21].

Female sex was also significantly associated with refusal of adjuvant therapy. Sex-specific differences in health-related decision-making, risk perception, and patient–physician communication have been described in population-based studies [22,23]. However, robust evidence addressing sex-related refusal patterns specifically in OSCC is limited, and this finding should therefore be interpreted with caution. Because no psychosocial, socioeconomic, or behavioral variables were available, the present data do not allow conclusions regarding the mechanisms underlying the observed sex-associated difference in treatment refusal.

In contrast, tumor-related variables, including pT and pN classification, were not significantly associated with refusal of adjuvant therapy. This suggests that treatment refusal was more closely associated with patient-level characteristics than with tumor stage in this cohort. However, this interpretation is limited by incomplete availability of selected pathological variables and by the absence of detailed comorbidity and frailty data. Similar observations have been reported in other oncologic settings, where objective disease severity did not reliably predict completion of recommended adjuvant treatment [24]. These findings underscore the importance of patient-centered counseling strategies that clearly communicate oncologic risk and expected benefit, particularly in patients with advanced disease.

Survival outcomes observed in this study are consistent with previously published data on advanced-stage OSCC, reporting 5-year OS rates between 30% and 50% [25,26]. The comparatively poor outcomes in the present cohort can largely be attributed to the high proportion of patients with advanced disease and adverse pathological features, reflecting the predefined inclusion criteria, as also reflected in contemporary reviews of head and neck cancer [4]. Subgroup analyses demonstrated inferior survival in patients with advanced pT and pN classification, in line with established prognostic models [27,28]. The present findings also align with larger registry-based analyses indicating that omission or refusal of recommended oncologic treatment is associated with worse outcomes, while emphasizing that registry and single-center cohorts differ substantially in selection mechanisms, treatment documentation, and available confounder adjustment.

From a clinical perspective, the present data highlight refusal of adjuvant therapy as a clinically relevant factor that may be modifiable through structured counseling and supportive care in the management of high-risk OSCC. However, given the observational design, causal relationships cannot be established. Early identification of patients at risk for refusal, particularly elderly patients, may facilitate targeted counseling and supportive interventions. Integration of geriatric assessment, psycho-oncologic support, and shared decision-making frameworks may help address treatment-related concerns and improve adherence to guideline-recommended therapy [29,30]. Such approaches may be particularly important for patients with advanced pathological risk features, in whom the expected benefit of adjuvant treatment is greatest.

Several limitations should be acknowledged. The retrospective single-center design limits generalizability and does not allow causal inference. In addition, the observed associations between completion of adjuvant therapy and survival outcomes may have been influenced by confounding by indication, immortal time bias, and unmeasured differences in comorbidity burden, frailty, ECOG performance status, treatment tolerance, socioeconomic factors, social support, and psychosocial variables that could not be adequately captured in this retrospective dataset.

The sample size was limited, which reduced statistical power, particularly in subgroup analyses, and may explain why some comparisons did not reach statistical significance despite consistent directional trends. No formal a priori sample size calculation was performed because this was a retrospective cohort study including all eligible patients treated during the predefined study period. Cox regression analyses were exploratory and restricted to a limited number of variables to avoid model overfitting; therefore, these results should be interpreted with caution. The complete-case restriction in multivariable models may have introduced additional selection bias. For DFS, Cox regression was not performed because the proportional hazards assumption was not met, indicating time-dependent effects that would violate model assumptions. Although alternative time-dependent modeling approaches could theoretically be considered, the limited sample size and subgroup event numbers were not sufficient for robust modeling. Furthermore, the absence of systematically documented reasons for treatment refusal limited the assessment of potentially relevant clinical and patient-related confounders.

Nevertheless, the homogeneity of the cohort, defined by a clearly established indication for adjuvant therapy, represents a key strength of this study and allowed a focused analysis of treatment refusal in a well-characterized high-risk population. All included patients had a definitive interdisciplinary tumor board recommendation for postoperative adjuvant treatment, reducing heterogeneity in treatment indication. While this applies to the OS analyses based on the full cohort, DFS analyses depended on available recurrence and follow-up documentation and should therefore be interpreted with appropriate caution. Overall, the findings support the clinical relevance of treatment adherence in high-risk OSCC, while underscoring the need for larger multicenter studies with standardized assessment of comorbidity, frailty, treatment preferences, and reasons for refusal.

## 5. Conclusions

This retrospective cohort study suggests that refusal of guideline-recommended adjuvant therapy represents a frequent clinical challenge in patients with high-risk OSCC and was associated with less favorable survival patterns, including shorter median OS and DFS in this cohort. Higher refusal rates were observed among elderly and female patients, although the underlying reasons for treatment refusal could not be systematically assessed. Survival outcomes consistently favored patients who completed guideline-recommended adjuvant therapy, although not all comparisons reached statistical significance and the analyses were exploratory in nature. Therefore, the observed associations should be interpreted as associative rather than causal. Overall, the findings support the potential clinical relevance of adherence to guideline-recommended postoperative therapy and underscore the importance of structured, patient-centered counseling strategies to support informed decision-making and improve treatment adherence in high-risk OSCC.

## Figures and Tables

**Table 1:** Distribution of primary tumor sites in the study cohort (n = 88).

Tumor Site	n (%)
Tongue	19 (21.6)
Mandibular gingiva	27 (30.7)
Maxillary gingiva	2 (2.3)
Floor of mouth	26 (29.5)
Palate	5 (5.7)
Buccal mucosa	9 (10.2)

**Table 2:** Association between patient age and refusal of adjuvant therapy (n = 88; *p* = 0.002).

Age Group	Refused (n, %)	Completed (n, %)	Total (n)
<50	1 (20.0%)	4 (80.0%)	5
50–59	2 (13.3%)	13 (86.7%)	15
60–69	6 (19.4%)	25 (80.6%)	31
70–79	7 (33.3%)	14 (66.7%)	21
80–89	11 (73.3%)	4 (26.7%)	15
>90	0 (0.0%)	1 (100.0%)	1

**Table 3:** Association between patient sex and refusal of adjuvant therapy (n = 88; *p* = 0.002).

Sex	Refused (n, %)	Completed (n, %)	Total (n)
Male	11 (19.6%)	45 (80.4%)	56
Female	16 (50.0%)	16 (50.0%)	32

**Table 4:** Association between primary tumor stage (pT) or nodal stage (pN) and refusal of adjuvant therapy (n = 88). Percentages are based on available cases with complete pathological staging data for the respective variable.

Variable	Refused n (%)	Completed n (%)	*p*-Value
pT1/2	9 (34.6)	17 (65.4)	0.869
pT3/4	9 (29.0)	22 (71.0)	
pN0/1	13 (34.2)	25 (65.8)	0.226
pN2/3	9 (20.0)	36 (80.0)	

**Table 5:** Median overall survival according to adjuvant therapy status (n = 88; survival comparison *p* = 0.155).

Adjuvant Therapy	Median OS (Months)	95% CI
Refused	33.8	not estimable
Completed	56.6	not estimable

Note: Median overall survival was estimated from Kaplan–Meier analysis. Upper confidence limits were not estimable in either group due to censoring.

**Table 6:** Exploratory Cox Proportional Hazards Models for Overall Survival. Bold text denotes the different Cox regression models included in the analysis.

Variable	Hazard Ratio (HR)	95% CI	*p*-Value
**Model 1** (univariate, n = 88)			
Adjuvant therapy (yes vs. no)	0.50	0.24–1.02	0.055
**Model 2** (adjusted for age and sex, n = 66)			
Adjuvant therapy (yes vs. no)	0.22	0.09–0.54	0.001
Age (per 10-year increase)	1.09	0.82–1.45	0.54
Sex (female vs. male)	1.31	0.72–2.39	0.38
**Model 3** (adjusted for age and nodal status, n = 66)			
Adjuvant therapy (yes vs. no)	0.23	0.09–0.57	0.002
Age (per 10-year increase)	0.96	0.66–1.41	0.85
pN2/3 vs. pN0/1	1.15	0.53–2.50	0.73

Note: Models 2 and 3 are based on complete-case analysis and should be interpreted as exploratory.

## Data Availability

The data presented in this study are available on request from the corresponding author. The data are not publicly available due to ethical and data protection restrictions.

## Abbreviations

OSCC	Oral squamous cell carcinoma.
DFS	Disease-free survival.
OS	Overall survival.
AT	Adjuvant therapy.

## References

[1] Rivera C. (2015). Essentials of Oral Cancer. Int. J. Clin. Exp. Pathol..

[2] Warnakulasuriya S. (2009). Global Epidemiology of Oral and Oropharyngeal Cancer. Oral Oncol..

[3] Choi S., Myers J.N. (2008). Molecular Pathogenesis of Oral Squamous Cell Carcinoma: Implications for Therapy. J. Dent. Res..

[4] Chow L.Q.M. (2020). Head and Neck Cancer. N. Engl. J. Med..

[5] Bernier J., Domenge C., Ozsahin M., Matuszewska K., Lefèbvre J.-L., Greiner R.H., Giralt J., Maingon P., Rolland F., Bolla M. (2004). Postoperative Irradiation with or without Concomitant Chemotherapy for Locally Advanced Head and Neck Cancer. N. Engl. J. Med..

[6] Cooper J.S., Pajak T.F., Forastiere A.A., Jacobs J., Campbell B.H., Saxman S.B., Kish J.A., Kim H.E., Cmelak A.J., Rotman M. (2004). Postoperative Concurrent Radiotherapy and Chemotherapy for High-Risk Squamous-Cell Carcinoma of the Head and Neck. N. Engl. J. Med..

[7] Pfister D.G., Spencer S., Adelstein D., Adkins D., Anzai Y., Brizel D.M., Bruce J.Y., Busse P.M., Caudell J.J., Cmelak A.J. (2020). Head and Neck Cancers, Version 2.2020, NCCN Clinical Practice Guidelines in Oncology. J. Natl. Compr. Cancer Netw..

[8] Fulcher C.D., Haigentz M., Ow T.J. (2018). The Education Committee of the American Head and Neck Society (AHNS) AHNS Series: Do You Know Your Guidelines? Principles of Treatment for Locally Advanced or Unresectable Head and Neck Squamous Cell Carcinoma. Head Neck.

[9] Pignon J.-P., Le Maître A., Maillard E., Bourhis J. (2009). Meta-Analysis of Chemotherapy in Head and Neck Cancer (MACH-NC): An Update on 93 Randomised Trials and 17,346 Patients. Radiother. Oncol..

[10] Mrosk F., Doll C., Scheer J., Neumann F., Hofmann E., Kreutzer K., Voss J., Rubarth K., Beck M., Heiland M. (2023). Oncologic Outcome in Advanced Oral Squamous Cell Carcinoma after Refusal of Recommended Adjuvant Therapy. JAMA Otolaryngol. Head Neck Surg..

[11] Schwam Z.G., Husain Z., Judson B.L. (2015). Refusal of Postoperative Radiotherapy and Its Association with Survival in Head and Neck Cancer. Radiother. Oncol..

[12] Tassone P., Topf M.C., Dooley L., Galloway T., Biedermann G., Trendle M. (2023). Going Off Guidelines: An NCDB Analysis of Missed Adjuvant Therapy Among Surgically Treated Oral Cavity Cancer. Otolaryngol.–Head Neck Surg..

[13] Kaanders J.H.A.M., van den Bosch S., Kleijnen J. (2022). Comparison of Patients With Head and Neck Cancer in Randomized Clinical Trials and Clinical Practice: A Systematic Review. JAMA Otolaryngol. Head Neck Surg..

[14] Leeman J.E., Li J., Pei X., Venigalla P., Zumsteg Z.S., Katsoulakis E., Lupovitch E., McBride S.M., Tsai C.J., Boyle J.O. (2017). Patterns of Treatment Failure and Postrecurrence Outcomes Among Patients With Locally Advanced Head and Neck Squamous Cell Carcinoma After Chemoradiotherapy Using Modern Radiation Techniques. JAMA Oncol..

[15] Herrmann T. (2015). Why Do Cancer Patients Refuse Curative Radiation Therapy?. Strahlenther. Onkol..

[16] Aizer A.A., Chen M.-H., Parekh A., Choueiri T.K., Hoffman K.E., Kim S.P., Martin N.E., Hu J.C., Trinh Q.-D., Nguyen P.L. (2014). Refusal of Curative Radiation Therapy and Surgery Among Patients With Cancer. Int. J. Radiat. Oncol..

[17] Gates C., Gillespie M., Kane A., Philips R., Curry J., Tekumalla S., Pettit C., Palmer T., Tassone P. (2025). Missed Adjuvant Therapy After Resection of Intermediate and High-Risk Oral Cavity Cancer: A Multi-Institutional Study. Otolaryngol.–Head Neck Surg..

[18] Wolff K.-D., Rau A., Weitz J., Langer T., Zidane M. (2021). Aktualisierung der S3-Leitlinie „Diagnostik und Therapie des Mundhöhlenkarzinoms“: Was ist neu?. MKG-Chirurg.

[19] Wolff K.-D., Bootz F., Beck J., Bikowski K., Böhme P., Budach W., Burkhardt A., Danker H., Eberhardt W., Engers K. (2012). Diagnostik Und Therapie Des Mundhöhlenkarzinoms.

[20] Sarasqueta C., Perales A., Escobar A., Baré M., Redondo M., Fernández De Larrea N., Briones E., Piera J.M., Zunzunegui M.V., Quintana J.M. (2019). Impact of Age on the Use of Adjuvant Treatments in Patients Undergoing Surgery for Colorectal Cancer: Patients with Stage III Colon or Stage II/III Rectal Cancer. BMC Cancer.

[21] Hamidi M., Moody J.S., Kozak K.R. (2010). Refusal of Radiation Therapy and Its Associated Impact on Survival. Am. J. Clin. Oncol..

[22] Mauvais-Jarvis F., Bairey Merz N., Barnes P.J., Brinton R.D., Carrero J.-J., DeMeo D.L., De Vries G.J., Epperson C.N., Govindan R., Klein S.L. (2020). Sex and Gender: Modifiers of Health, Disease, and Medicine. Lancet.

[23] Wagner A.D., Oertelt-Prigione S., Adjei A., Buclin T., Cristina V., Csajka C., Coukos G., Dafni U., Dotto G.-P., Ducreux M. (2019). Gender Medicine and Oncology: Report and Consensus of an ESMO Workshop. Ann. Oncol..

[24] Männle H., Siebers J.W., Momm F., Münstedt K. (2021). Impact of Patients’ Refusal to Undergo Adjuvant Treatment Measures on Survival. Breast Cancer Res. Treat..

[25] Eckert A.W., Lautner M.H.W., Dempf R., Schubert J., Bilkenroth U. (2009). Prognostische Aussagen zum Mundhöhlenkarzinom. Chirurg.

[26] Farhood Z., Simpson M., Ward G.M., Walker R.J., Osazuwa-Peters N. (2019). Does Anatomic Subsite Influence Oral Cavity Cancer Mortality? A SEER Database Analysis. Laryngoscope.

[27] Ong T.K., Murphy C., Smith A.B., Kanatas A.N., Mitchell D.A. (2017). Survival After Surgery for Oral Cancer: A 30-Year Experience. Br. J. Oral Maxillofac. Surg..

[28] Quinlan-Davidson S.R., Mohamed A.S., Myers J.N., Gunn G.B., Johnson F.M., Skinner H., Beadle B.M., Gillenwater A.M., Phan J., Frank S.J. (2017). Outcomes of Oral Cavity Cancer Patients Treated with Surgery Followed by Postoperative Intensity Modulated Radiation Therapy. Oral Oncol..

[29] Hamaker M.E., Vos A.G., Smorenburg C.H., Rooij S.E., Munster B.C. (2012). The Value of Geriatric Assessments in Predicting Treatment Tolerance and All-Cause Mortality in Older Patients with Cancer. Oncologist.

[30] Stacey D., Légaré F., Lewis K., Barry M.J., Bennett C.L., Eden K.B., Holmes-Rovner M., Llewellyn-Thomas H., Lyddiatt A., Thomson R. (2017). Decision Aids for People Facing Health Treatment or Screening Decisions. Cochrane Database Syst. Rev..

